# Utilisation patterns and exploratory short-term outcomes of drug-coated balloon versus drug-eluting stent in percutaneous coronary intervention: a single-centre observational cohort study

**DOI:** 10.1186/s43044-026-00796-7

**Published:** 2026-11-10

**Authors:** M. B. Faslur Rahuman, M. R. Mubarak, S. Mithrakumar, Sanjeewa Rajapakse, Reeni Robert, Keerthyga Thayaparan, Senil Visura, Pubudu De Silva

**Affiliations:** 1 Lanka Hospitals, Colombo, Sri Lanka; 2 Colombo South Teaching Hospital, Colombo, Sri Lanka; 3 Colombo North Teaching Hospital, Colombo, Sri Lanka; 4 Colombo South Teaching Hospital, Colombo, Sri Lanka; 5 Ministry of Health, Colombo, Sri Lanka

**Keywords:** Drug coated balloon, Drug eluting stent, Percutaneous coronary intervention, In stent restenosis

## Abstract

**Background:**

Drug-coated balloons (DCBs) are an established alternative to drug-eluting stents (DESs) for selected coronary lesions, offering the potential advantages of a stent-free strategy while avoiding permanent metallic implantation. This study compared real-world utilisation patterns, clinical outcomes, and patient-reported outcomes following percutaneous coronary intervention (PCI) with DCBs and DESs.

**Methods:**

This single-centre retrospective cohort study with prospective follow-up included 214 consecutive patients who underwent PCI with either a DCB (*n* = 64) or DES (*n* = 150) between January 2023 and March 2026. Baseline demographic, angiographic, procedural, and clinical characteristics were compared. Patient-reported outcomes were assessed at follow-up using a questionnaire adapted from the domains of the Seattle Angina Questionnaire. Multivariable logistic regression was performed to identify independent factors associated with DCB utilisation.

**Results:**

The study included 214 patients (mean age 56.55 ± 11.49 years), of whom 72.9% were male. Clinical presentation differed significantly between groups (*p* = 0.042), the ST-elevation myocardial infarction (STEMI) group more frequently underwent DES (46.0% vs. 29.7%), whereas non-ST-elevation myocardial infarction (NSTEMI) (37.5% vs. 30.7%) and Unstable Angina​ (UA) (15.6% vs. 6.0%) were more common in the DCB group. DCBs were preferentially used in patients with previous coronary revascularization, ISR, small-vessel disease and elective PCI, whereas DESs were more commonly used for acute PCI, total occlusive lesions, and lesions with calcification or thrombus (all *p* < 0.05). Previous coronary revascularization was associated with increased odds of DCB use, while acute PCI was associated with reduced odds of DCB use compared with elective PCI. No significant differences were observed between groups in mortality at 1, 3, or 6 months, other early clinical outcomes, or patient-reported outcomes.

**Conclusions:**

DCBs were preferentially used in specific clinical and anatomical settings while demonstrating low observed rates of short-term clinical events and no statistically significant between-group differences in patient-reported outcomes. Despite differences in lesion complexity and treatment selection, no significant differences in early outcomes were observed compared with DES. These findings describe contemporary real-world DCB utilisation in a Sri Lankan PCI centre and provide a basis for further multicentre evaluation.

## Introduction

Coronary artery disease (CAD) remains a leading cause of morbidity and mortality worldwide and is an increasingly important public health problem in Sri Lanka [1], driven by the rising prevalence of diabetes mellitus, hypertension, dyslipidaemia, and tobacco use [2, 3]. Percutaneous coronary intervention (PCI) is a cornerstone in the management of obstructive CAD, with drug-eluting stents (DES) being the most commonly used devices for coronary revascularization [4]. While contemporary DES platforms demonstrate excellent efficacy, their use involves permanent metallic implantation and the need for prolonged dual antiplatelet therapy (DAPT) [5, 6]. More recently, drug-coated balloons (DCBs) have emerged as an alternative revascularization strategy in selected lesion subsets, offering the potential for effective treatment without permanent intracoronary implantation [7].

In clinical practice, the selection between DES and DCBs is often influenced not only by lesion characteristics but also by patient-specific cardiovascular risk factors and overall disease burden. Despite advances in stent technology, certain clinical and anatomical scenarios remain challenging. These include small-vessel disease, in-stent restenosis (ISR), bifurcation lesions, diffuse disease, and situations where prolonged DAPT is undesirable [8]. In selected clinical and anatomical settings, avoidance of permanent metallic implantation may be desirable, particularly when minimizing metal burden or duration of antiplatelet therapy is an important consideration. DCB represent an alternative revascularization strategy that delivers an antiproliferative drug—either paclitaxel or sirolimus—to the vessel wall without leaving a permanent implant [9].

Although evidence supporting the safety and efficacy of DCBs is well established in Western populations, data from South Asian countries, including Sri Lanka, show a paucity of systematically collected local data describing indications, lesion patterns, procedural characteristics, and clinical outcomes of DCB angioplasty [10]. Therefore, extrapolation of international trial data to the Sri Lankan population may not be fully appropriate [11]. Furthermore, despite increasing use in routine clinical practice, DCB utilisation is limited by high costs and lack of availability in the state sector [12], resulting in restricted access and relatively small study populations, largely confined to single-centre institutional experiences.

However, understanding real-world practice is essential for evaluating the appropriateness of DCB use, optimizing patient selection, and guiding resource allocation in a low- to middle-income healthcare setting [13]. Furthermore, local outcome data may contribute to the regional evidence base and identify areas for quality improvement and future research [14]. This study aimed to describe DCB and DES utilisation patterns and compare short-term clinical and patient-reported outcomes among patients undergoing PCI at a Sri Lankan cardiac centre.

## Methods

### Study design and setting

This was a single-centre retrospective observational cohort study with prospective follow-up, conducted at a tertiary cardiac centre in Sri Lanka. Consecutive adult patients who underwent PCI with either DCB angioplasty or DES implantation by selected visiting cardiologists between January 2023 and March 2026.

The retrospective component comprised the collection of demographic, clinical, angiographic, procedural and in-hospital outcome data from institutional medical records and PCI databases. The prospective component consisted of clinical follow-up and collection of patient-reported outcomes (PROs) at predefined time points. Follow-up assessments were performed during the index hospitalisation and at 1, 3 and 6 months following PCI.

The study centre provides tertiary-level cardiac care and performs both elective and emergency PCI. DCB therapy was available during the entire study period. The decision to use DCB angioplasty or DES implantation was made by the treating interventional cardiologist based on the clinical presentation, angiographic characteristics and procedural considerations.

### Study population and eligibility criteria

Patients aged ≥ 18 years who underwent PCI using DCB angioplasty or DES implantation during the study period were eligible for inclusion. Both de novo coronary lesions and ISR lesions were eligible for inclusion.

**Fig. 1:**
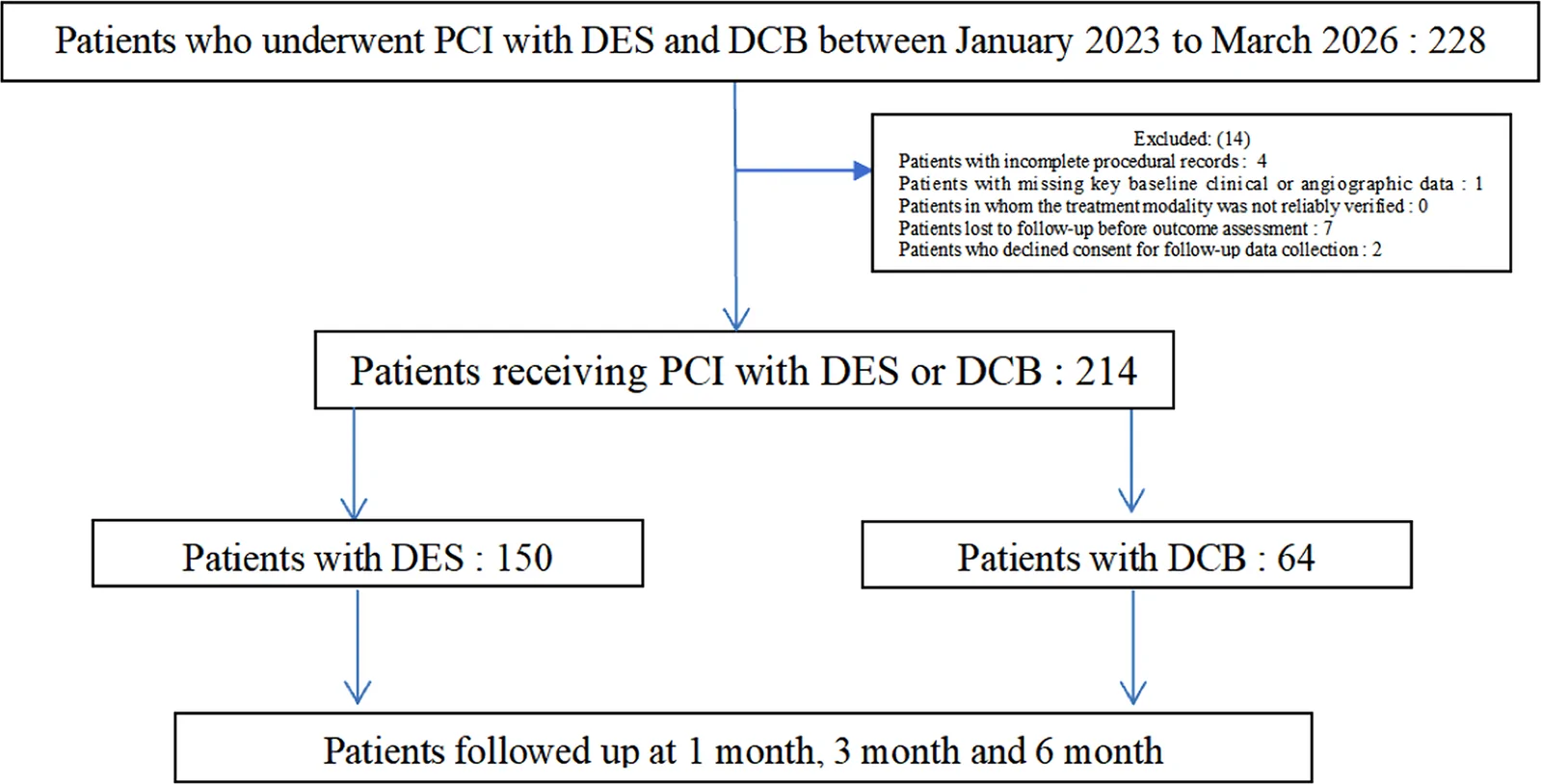
Flowchart of patient selection and follow-up

Patients with incomplete procedural records, missing key baseline clinical or angiographic data, inability to reliably verify the treatment modality, loss to follow-up before outcome assessment, or refusal to provide consent for follow-up data collection were excluded (Fig. 1).

The primary unit of analysis was the patient. Procedural and angiographic characteristics were additionally recorded at the lesion level where applicable.

Patients who received both DCB and DES during the same index PCI procedure were classified within the DCB group for the purposes of the comparative analysis.

### Baseline clinical and demographic characteristics

Baseline demographic and clinical characteristics were obtained from electronic medical records and PCI documentation. Variables included age, sex, cardiovascular risk factors, previous CAD, previous myocardial infarction, previous PCI or coronary artery bypass grafting (CABG), hypertension, diabetes mellitus, dyslipidaemia, smoking status, chronic kidney disease (CKD) and other relevant comorbidities.

The clinical presentation at the time of PCI was recorded according to the documented clinical diagnosis and included stable coronary disease and acute coronary syndromes (ACS), where applicable.

### Angiographic and procedural assessment

Angiographic and procedural characteristics were obtained from the PCI report and available coronary angiographic records. Variables included target vessel, lesion location, lesion morphology, indication for PCI, lesion preparation strategy, number and type of DCBs or DESs used, and procedural complications.

Coronary vessels were classified as small or large according to visual assessment of angiographic vessel calibre by the treating or interpreting cardiologist. No specific millimetre threshold or quantitative coronary angiographic diameter measurement was used in this study. In resource-constrained settings where intracoronary imaging modalities are not routinely available, vessel calibre may be assessed by visual angiographic estimation or, where available, quantitative coronary angiography [15].

Coronary disease was classified as focal when confined to a discrete, well-defined stenotic segment and as diffuse when involving a long segment of the vessel with extensive or continuous luminal narrowing rather than a single focal lesion. Classification of focal versus diffuse disease was based on visual angiographic assessment by the treating cardiologist [16].

Where angiographic characteristics were not explicitly documented, they were recorded as not documented/missing and were not inferred from other clinical or procedural variables.

Left ventricular ejection fraction (EF) was obtained from the most recent echocardiographic assessment available before or during the index admission. EF was categorized into three predefined groups: <30%, 30–55%, and > 55%, consistent with classifications used in previous cardiovascular studies [17, 18]. Where a quantitative EF measurement was unavailable, the value was recorded as missing.

### DCB and DES treatment

The decision to perform DCB angioplasty or implant a DES was made by the treating interventional cardiologist according to the clinical and angiographic characteristics of the target lesion and the intended PCI strategy.

For DCB-treated lesions, lesion preparation was performed using balloon angioplasty or other appropriate lesion-preparation techniques before DCB delivery. The DCB was subsequently positioned across the target lesion and inflated according to the device-specific technique and operator judgement. An angiographic assessment was performed following DCB treatment to determine the adequacy of the procedural result.

DES implantation was performed according to conventional PCI practice, with lesion preparation, stent selection and post-dilatation undertaken according to lesion characteristics and operator judgement.

ISR was defined as recurrent coronary stenosis involving a previously implanted stent or its immediate margins. ISR lesions were identified from the angiographic and procedural documentation. The location and characteristics of ISR lesions were recorded where available.

### Clinical outcomes and follow-up

Clinical outcomes were assessed during the index hospitalisation and at 1, 3 and 6 months following PCI. Outcomes assessed included all-cause mortality, cardiac mortality, and major cardiac and non-cardiac adverse events. Cardiac events included ST-elevation myocardial infarction (STEMI), non-ST-elevation myocardial infarction (NSTEMI), and heart failure (HF). Non-cardiac events included stroke, acute kidney injury (AKI), bleeding, and sepsis. Cardiac mortality was defined as death due to any cardiac cause, including heart failure. Heart-failure-related mortality was specifically defined as death attributed to heart failure. Mortality status and cause of death were determined from follow-up assessments and available hospital records.

Target lesion revascularisation (TLR) was defined as repeat revascularisation involving the previously treated target lesion, while target vessel revascularisation (TVR) was defined as repeat revascularisation involving the previously treated target vessel.

Follow-up information was obtained through outpatient clinic visits, telephone interviews and hospital records. Where direct follow-up information was unavailable, available institutional records were reviewed to ascertain clinical events.

### Patient-reported outcomes

PROs were assessed prospectively by the interviewer using four questions derived from the Seattle Angina Questionnaire (SAQ), addressing angina frequency, physical limitation, enjoyment of life, and treatment satisfaction. The questions were administered at 1, 3 and 6 months following the index PCI. Each domain was assessed using a four-point ordinal response scale, with responses graded as 1 (very poor), 2 (poor), 3 (good), and 4 (excellent), with higher scores representing more favourable patient-reported status [19].

PRO analyses were restricted to patients who were alive and had completed the relevant follow-up assessment. Accordingly, patients who died before a given PRO assessment were not included in the corresponding PRO analysis. At the 6-month assessment, for example, PRO data were available for 61 patients in the DCB group and 147 patients in the DES group, reflecting the patients who remained alive and had available PRO responses at that time point.

Because the study used four questions derived from the SAQ rather than the complete validated SAQ instrument, the responses were analysed as individual patient-reported domains and were not interpreted as a validated overall SAQ summary score.

### Statistical analysis

Statistical analyses were performed using SPSS version 25.0 and STATA version 19. Continuous variables were assessed for distribution and are presented as mean ± standard deviation (SD) for approximately normally distributed variables or median with interquartile range (IQR) for non-normally distributed variables. Categorical variables are presented as frequencies and percentages.

Comparisons between the DCB and DES groups were performed using the independent-samples *t* test for normally distributed continuous variables and the Mann–Whitney *U* test for non-normally distributed continuous variables and ordinal variables. Categorical variables were compared using the chi-square test or Fisher’s exact test, as appropriate. Kaplan–Meier survival analysis was performed to estimate overall survival probabilities during the 6-month follow-up period. Survival status was assessed at the scheduled 1-, 3-, and 6-month follow-up visits, and survival time was assigned according to the follow-up interval at which the event was identified. Patients who remained alive at the end of follow-up were censored at their last available follow-up assessment. Survival distributions between the DCB and DES groups were compared using the log-rank test. Patient-reported outcome domains were treated as ordinal variables and are presented as median (IQR). Comparisons of the four PRO domains between the DCB and DES groups at each follow-up assessment were performed using the Mann–Whitney *U* test. PRO analyses included only patients who were alive and had available responses at the respective assessment time point. Missing PRO responses among surviving patients were not imputed.

All statistical tests were two-sided, and a *p* value < 0.05 was considered statistically significant.

## Results

A total of 214 patients fulfilled the study inclusion criteria. Among these, 64 patients were treated with a DCB and 150 patients underwent DES. The patient selection process, including exclusions and the derivation of the final study cohort, is presented in Fig. 1.

### Baseline and clinical presentation

Table 1 summarizes the baseline characteristics and clinical presentation of patients according to treatment modality. ​The mean age was 56.55 ± 11.49 in the DCB group and 57.94 ± 10.58 in the DES group. The majority of patients were male in both the DCB and DES groups (76.6% vs. 71.3%, *p* = 0.431).

There were no significant differences in individual cardiovascular risk factors or EF. However, the overall distribution of clinical presentation differed significantly between treatment groups (*p* = 0.042). Patients treated with DES more frequently presented with STEMI (46.0% vs. 29.7%), whereas NSTEMI (37.5% vs. 30.7%) and unstable angina (UA) (15.6% vs. 6.0%) were relatively more common among patients treated with DCB.

**Table 1:** Baseline characteristics and clinical presentation

Variable	DCB (*n* = 64)	DES (*n* = 150)	*p*-value
Age, years	56.55 ± 11.49	57.94 ± 10.58	
Male sex, n (%)	49 (76.6)	107 (71.3)	0.431
Female sex, n (%)	15 (23.4)	43 (28.7)	
Dyslipidemia, n (%)	38 (59.4)	109 (72.7)	0.055
Hypertension, n (%)	31 (48.4)	84 (56.0)	0.310
Diabetes mellitus, n (%)	36 (56.3)	84 (56.0)	0.973
Chronic kidney disease, n (%)	5 (7.8)	8 (5.3)	0.536​
Family history of IHD, n (%)	6 (9.4)	28 (18.7)	0.089
Clinical presentation, n (%)			0.042
• STEMI	19 (29.7)	69 (46.0)	
• NSTEMI	24 (37.5)	46 (30.7)	
• UA	10 (15.6)	9 (6.0)	
• Stable angina	11 (17.2)	26 (17.3)	
Ejection fraction, n (%)			0.242
• >55%	20 (31.3)	43 (28.7)	
• 30–55%	43 (67.2)	96 (64.0)	
• <30%	1 (1.6)	11 (7.3)	

IHD ischemic heart disease, STEMI ST-elevation myocardial infarction, NSTEMI non-ST-elevation myocardial infarction, UA unstable angina

### Angiographic and procedural characteristics

Table 2 summarizes the procedural and angiographic characteristics according to treatment modality. Patients treated with DCB were significantly more likely to have undergone previous coronary revascularization than those treated with DES (34.4% vs. 5.3%, *p* < 0.001). Consistent with contemporary practice, all lesions presenting with ISR were treated with DCB, whereas no ISR lesions were managed with DES (*p* < 0.001).

Significant differences were also observed in lesion and procedural characteristics. DCBs were preferentially used for small-vessel disease and branch-vessel lesions, whereas DESs were predominantly implanted in large main-vessel disease (*p* < 0.001 for both comparisons). Same day/Acute PCI was performed more frequently in the DES group (86.7% vs. 71.9%), while delayed/elective PCI was more common among patients treated with DCB (*p* = 0.010). In addition, lesion calcification and intracoronary thrombus were observed among DES-treated lesions (*p* < 0.001 for both).

**Table 2:** Angiographic and procedural characteristics

Variable	DCB (*n* = 64)	DES (*n* = 150)	*P* value
Extent of coronary artery disease, n (%)			0.269
• Single-vessel disease	32 (50.0)	71 (47.3)	
• Double-vessel disease	19 (29.7)	59 (39.3)	
• Triple-vessel disease	13 (20.3)	20 (13.3)	
Previous revascularization, n (%)			< 0.001*
• None	42 (65.6)	142 (94.7)	
• Previous PCI	20 (31.3)	8 (5.3)	
• Previous PCI + CABG	2 (3.1)	0 (0.0)	
In-stent restenosis at presentation, n (%)	12 (18.8)	0 (0.0)	< 0.001*
Lesion characteristics, n (%)			0.025*
• Discrete	2 (3.1)	0 (0.0)	
• Diffuse	12 (18.8)	17 (11.3)	
• Not specified	50 (78.1)	133 (88.7)	
Vessel size, n (%)			< 0.001*
• Small vessel disease	9 (14.1)	0 (0.0)	
• Large vessel disease	55 (85.9)	150 (100.0)	
Target vessel location, n (%)			< 0.001*
• Main vessel disease	37 (57.8)	139 (92.7)	
• Branch vessel disease	12 (18.8)	0 (0.0)	
• Main + branch vessel disease	15 (23.4)	11 (7.3)	
Timing of PCI, n (%)			0.010*
• Same day/ Acute	46 (71.9)	130 (86.7)	
• Delayed/ Elective	18 (28.1)	20 (13.3)	
Number of vessels treated, n (%)			0.099
• Single-vessel PCI	38 (59.4)	99 (66.0)	
• Double-vessel PCI	20 (31.3)	47 (31.3)	
• Triple-vessel PCI	6 (9.4)	4 (2.7)	
Lesion severity, n (%)			0.053
• 70–99% stenosis	54 (84.4)	108 (72.0)	
• Total Occlusion	10 (15.6)	42 (28.0)	
Arterial access, n (%)			0.028*
• Radial	61 (95.3)	150 (100.0)	
• Femoral	2 (3.1)	0 (0)	
• Ulnar	1 (1.6)	0 (0)	
Additional lesion characteristics, n (%)			< 0.001*
• Not specified	64 (100.0)	116 (77.3)	
• Calcification	0 (0)	10 (6.7)	
• Thrombus	0 (0)	24 (16.0)	
Procedural complications, n (%)			0.125
• Type B dissection	1 (1.6)	0 (0.0)	

PCI percutaneous coronary intervention, CABG coronary artery bypass graft

### Coronary vessel distribution

The distribution of treated coronary vessels according to Device strategy is presented in Table 3. Among patients undergoing PCI, single-vessel intervention was the most common strategy in both groups, with the left anterior descending artery (LAD) being the most frequently treated vessel, particularly in the DES group (51.3%). Double-vessel PCI was also commonly performed, most often involving the LAD and the right coronary artery (RCA) combination, while several branch vessel combinations (e.g., D1, OM, RI, and PDA) were observed predominantly in the DCB group.

**Table 3:** Distribution of treated coronary vessels according to device strategy

PCI category	DCB (*n* = 64)	DES (*n* = 150)
Single-vessel PCI		
LAD	14 (21.9)	77 (51.3)
RCA	10 (15.6)	24 (16.0)
LCX	4 (6.3)	2 (1.3)
D1	1 (1.6)	0 (0)
OM1	2 (3.1)	0 (0)
PLV	1 (1.6)	0 (0.0%)
RPDA	1 (1.6)	0 (0.0)
RI	2 (3.1)	0 (0.0)
Double-vessel PCI		
LAD + LCX	3 (4.7)	11 (7.3)
RCA + LCX	3 (4.7)	3 (2.0)
LAD + RCA	7 (10.9)	27 (18.0)
D1 + OM1	2 (3.1)	0 (0.0)
LCX + OM1	3 (4.7)	1 (0.7)
LAD + D1	3 (4.7)	0 (0)
PDA + OM3	1 (1.6)	0 (0)
LAD + OM1	0 (0)	1 (0.7)
LMCA + RCA	0 (0)	1 (0.7)
Triple-vessel PCI		
LAD + LCX + RCA	5 (7.8)	2 (1.3)
OM2 + D1 + LAD	1 (1.6)	0 (0)
RCA + LAD + OM1	0 (0)	1 (0.7)
LCX + RCA + RI	1 (1.6)	0 (0)

LAD left anterior descending, RCA right coronary artery, LCX left circumflex, D1 diagonal, OM1 obtuse marginal, PLV posterolateral ​ventricular branch, RPDA right posterior descending artery, RI ramus intermedius, LMCA left main coronary arteryp

### Multivariable logistic regression analysis

**Fig. 2:**
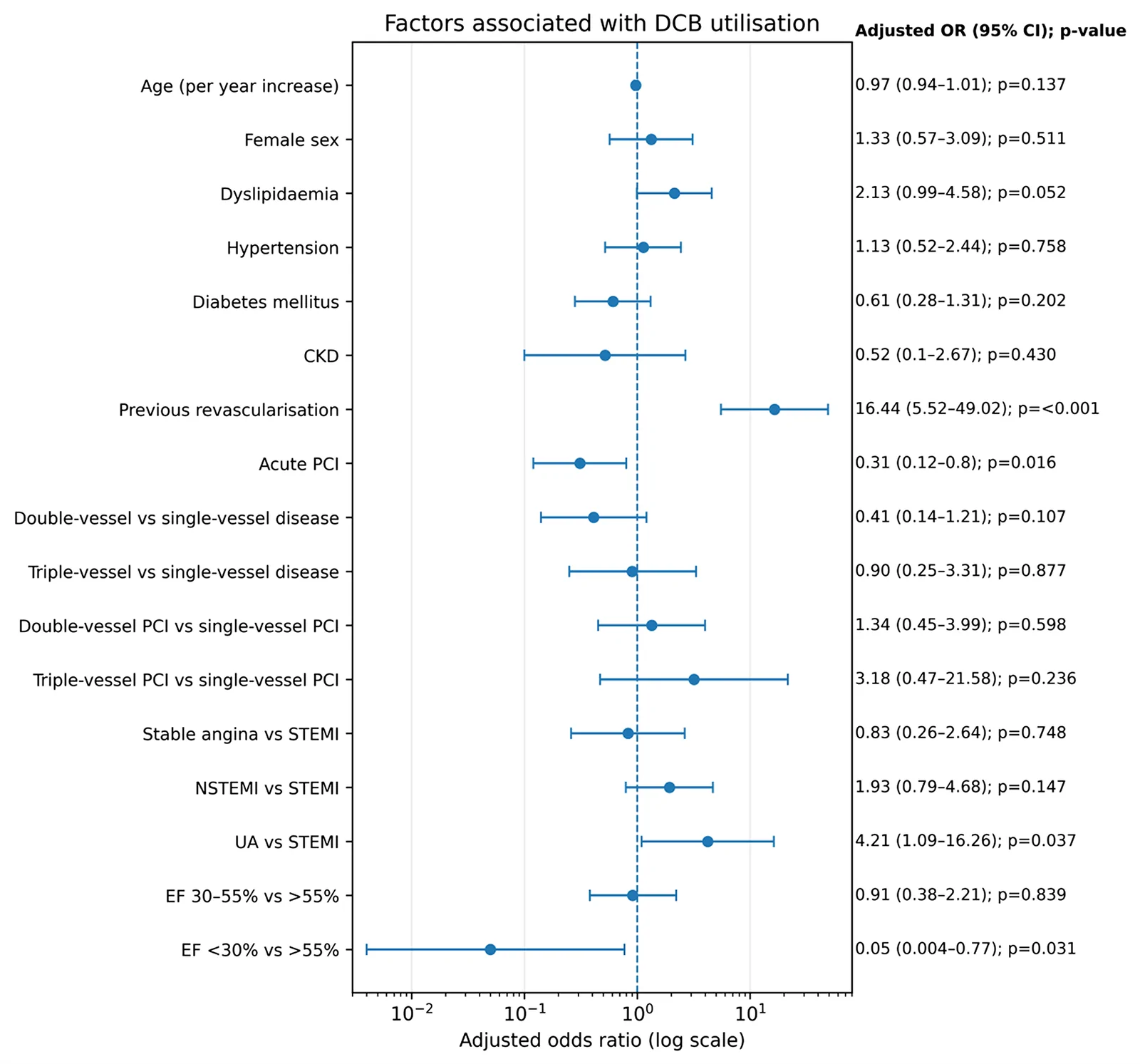
Forest plot of factors associated with DCB utilisation based on multivariable logistic regression analysis. CKD chronic kidney disease, PCI percutaneous coronary intervention, STEMI ST elevation myocardial infarction, NSTEMI Non-ST-elevation myocardial infarction, UA unstable angina. Reference categories: Male sex, no dyslipidaemia, no hypertension, no diabetes, no CKD, no previous revascularization, elective PCI, single vessel disease, single vessel PCI, STEMI, ejection fraction > 55%

A multivariable logistic regression model was performed to identify factors associated with DCB use compared with DES (Fig. 2). Although the number of DCB cases (*n* = 64) limited the number of covariates that could be reliably assessed, the model was considered appropriate for exploratory evaluation of treatment-selection patterns, with findings interpreted cautiously. The observed associations were assessed in the context of clinical plausibility and consistency with established DCB utilisation patterns.

Previous revascularization was independently associated with increased odds of DCB use (adjusted OR 16.44, 95% CI 5.52–49.02, *p* < 0.001). This finding is biologically plausible and consistent with current DCB indications, particularly for patients with prior PCI and ISR [20]. Conversely, acute PCI was associated with reduced odds of DCB use compared with elective PCI (adjusted OR 0.31, 95% CI 0.12–0.80, *p* = 0.016). This finding is consistent with contemporary clinical practice, where DES remain the predominant revascularization strategy in ACS when stent implantation is required, supported by extensive evidence and guideline recommendations. In contrast, DCB therapy has been primarily adopted for elective indications such as ISR, small-vessel disease, and selected de novo lesions.

Among the different clinical presentations, UA was independently associated with higher odds of DCB use compared with STEMI (adjusted OR 4.21, 95% CI 1.09–16.26; *p* = 0.037), whereas NSTEMI was not significantly associated with DCB use compared with STEMI (adjusted OR 1.93, 95% CI 0.79–4.68; *p* = 0.147). The association observed with UA should be interpreted cautiously given the relatively wide confidence interval, suggesting limited precision of the estimate.

Other demographic and angiographic variables were not significantly associated with device selection. Patients with EF < 30% had lower odds of receiving DCB compared with those with EF > 55% (adjusted OR 0.05, 95% CI 0.004–0.77, *p* = 0.031); however, this is an unstable estimate and this association should be interpreted cautiously because of the small number of patients within the severely reduced EF subgroup and the possibility of model instability. Overall, the significant associations observed in this analysis were clinically coherent and likely reflect real-world decision-making regarding device selection; however, given the observational single-centre design and limited sample size, these findings should be considered hypothesis-generating rather than causal.

### Clinical outcomes

In Table 4, clinical outcomes at 6 months were infrequent in both treatment groups, with no statistically significant differences observed. All-cause mortality was 4.7% in the DCB group and 2.0% in the DES group (*p* = 0.367). All cardiac deaths in both groups were attributed to heart failure, accounting for two deaths in the DCB group (3.1%) and two in the DES group (1.3%; *p* = 0.585). One non-cardiac death occurred in each group, due to a cerebrovascular event and sepsis, respectively. A single non-fatal cardiac event occurred in the DCB group (1.6%; *p* = 0.299). Among the four patients whose deaths were attributed to heart failure, three had a recorded LVEF of 30–55%, while one had an LVEF of < 30.

**Table 4:** Cumulative all-cause mortality by 6 months

Outcome	DCB (*n* = 64)	DES (*n* = 150)	*p* value
Mortality at 1 month	2 (3.1%)	2 (1.3%)	0.585
Mortality at 3 months	3 (4.7%)	2 (1.3%)	0.159
Mortality at 6 months	3 (4.7%)	3 (2.0%)	0.367
Cardiac mortality	2 (3.1%)	2 (1.3%)	0.585
└─ Heart failure-related mortality	2 (3.1%)	2 (1.3%)	-
Non-cardiac mortality	1 (1.6%)	1 (0.7%)	0.510
└─ Cause of non-cardiac mortality	Cerebrovascular event (CVA): 1 (1.6%)	Sepsis: 1 (0.7%)	-
Cardiac events during follow-up (non-fatal)	1 (1.6%)	0 (0%)	0.299

Values are presented as n (%). Fisher’s exact test was used due to low event counts

**Fig. 3:**
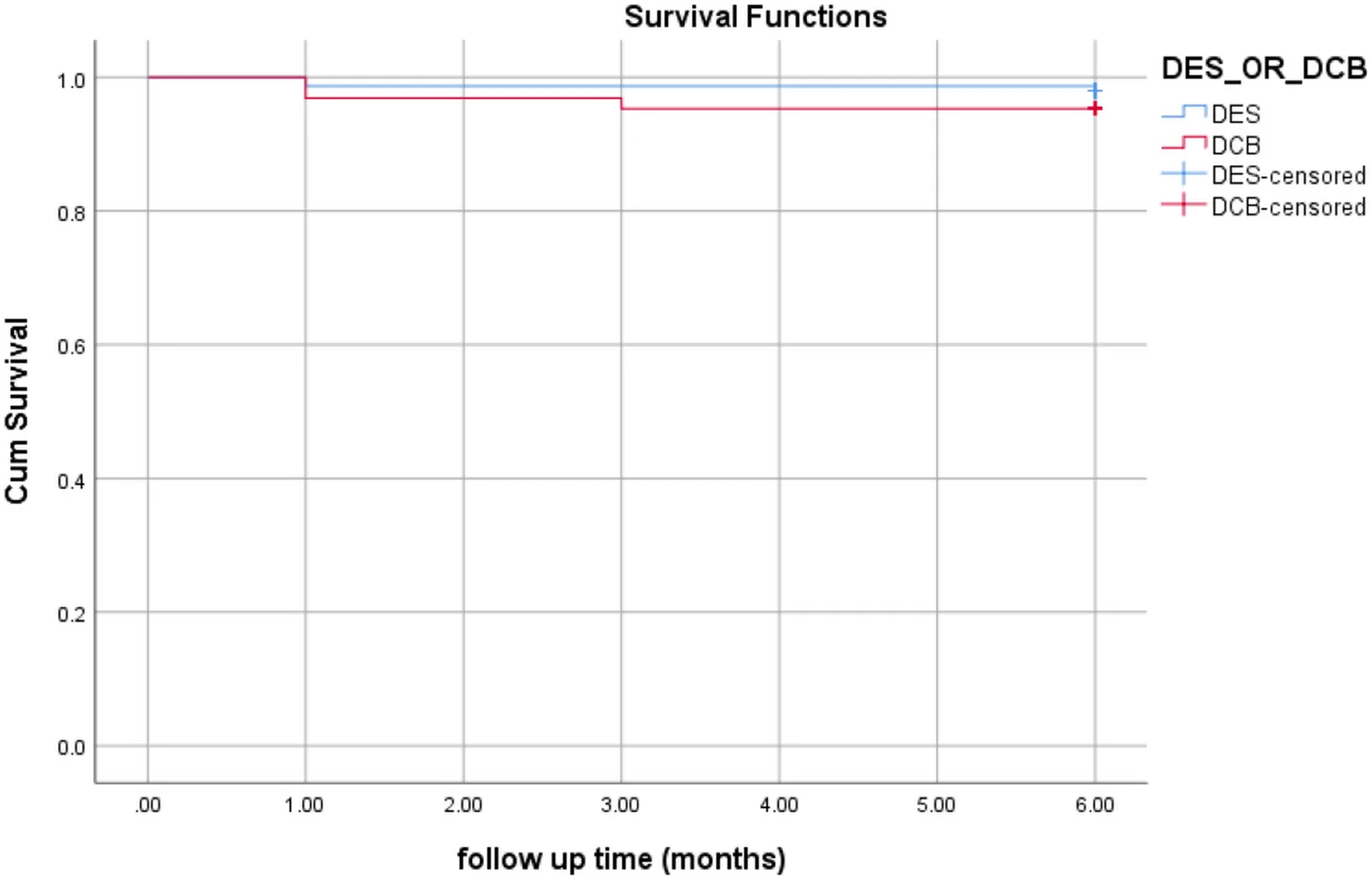
Kaplan–meier analysis of overall survival after DCB vs. DES treatment

There was no statistically significant difference in 6-month overall survival between the DCB and DES groups (log-rank test: χ²=1.192, *p* = 0.275) (Fig. 3). Few mortality events were observed, and event times were recorded based on follow-up assessments rather than exact dates of death; therefore, Kaplan–Meier estimates and log-rank comparisons should be interpreted with caution.

### Patient-reported symptom and functional status outcomes at follow-up

Patient-reported symptom and functional status measures were obtained from all 208 patients who survived to follow-up in Table 5. Overall, PROs were favourable in both treatment groups, with high scores across all assessed domains. There were no significant differences between the DES and DCB groups in angina frequency, physical limitation, enjoyment of life, or treatment satisfaction, indicating comparable patient-reported symptom and functional status at follow-up.

**Table 5:** Patient-reported symptom and functional status outcomes at follow-up according to device strategy

Patient-reported outcome domain	DCB (*n* = 61) Median (IQR)	DES (*n* = 147) Median (IQR)	*p*-value*
Angina frequency	4 (4–4)	4 (4–4)	0.539
Physical limitation	4 (4–4)	4 (4–4)	0.566
Enjoyment of life	4 (4–4)	4 (4–4)	0.212
Treatment satisfaction	4 (4–4)	4 (4–4)	0.488

*Data are presented as median (IQR). Only patients who were alive and had available PRO responses at 6 months were included. p-values were calculated using the Mann–Whitney U test

**Fig. 4:**
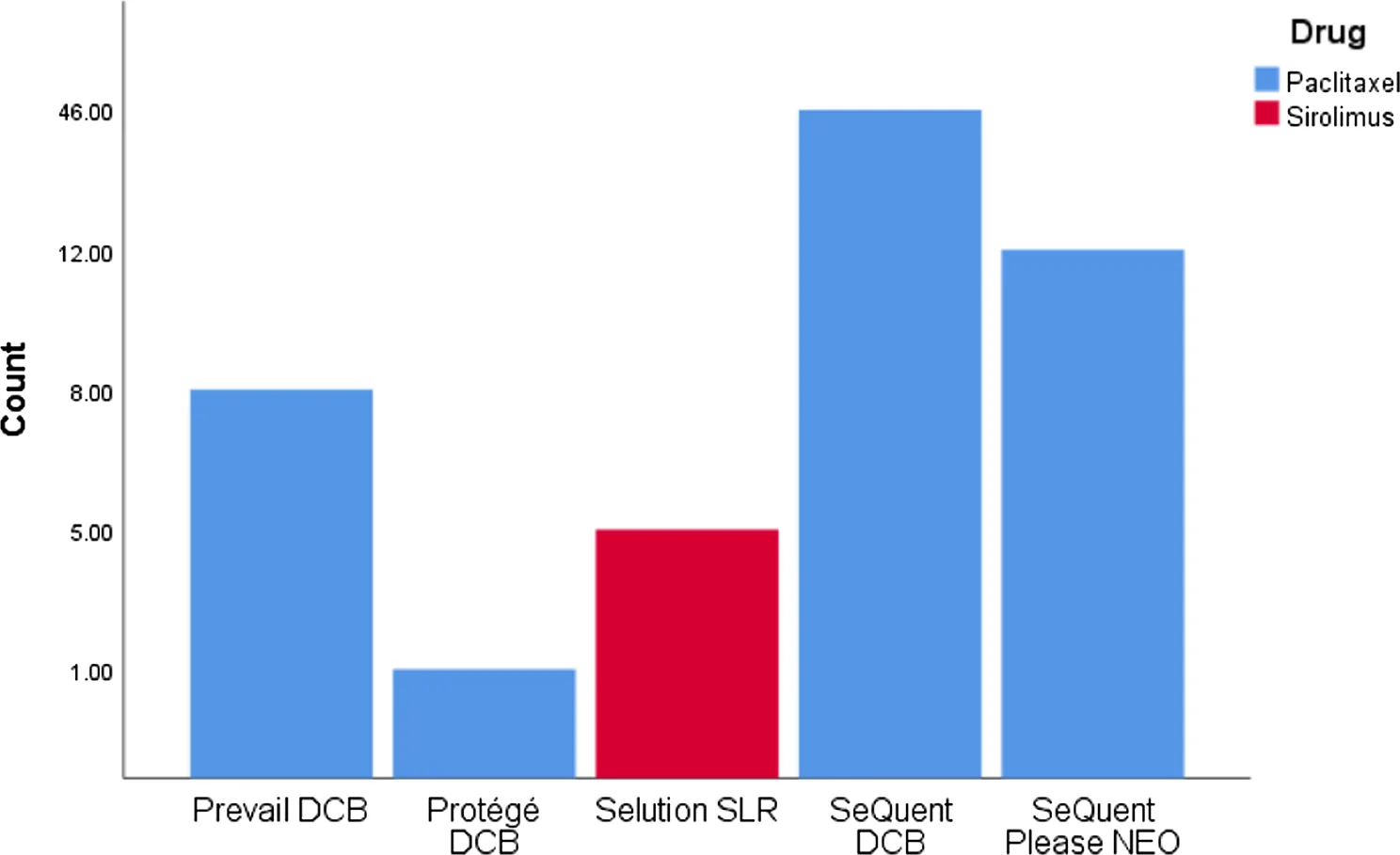
Distribution of DCB brands according to antiproliferative drug used

Among the 64 patients treated with DCB, a total of 73 DCBs were used. This difference reflects the use of more than one DCB in some patients when multiple lesions or coronary segments required treatment. SeQuent DCB was the most frequently utilized brand (*n* = 46, 63.0%), followed by SeQuent Please NEO DCB (*n* = 12, 16.4%) and Prevail DCB (*n* = 8, 11.0%). Selution SLR DCB was the only sirolimus-coated balloon used (*n* = 5, 6.8%), whereas all other brands were paclitaxel-coated balloons. Consequently, paclitaxel-coated balloons accounted for 93.2% of all DCBs used in the study, illustrated in Fig. 4. The limited use of sirolimus-coated balloons likely reflects their relatively limited adoption and availability in our setting during the study period.

## Discussion

### Principal findings and contemporary DCB utilisation

In this single-centre retrospective observational study, we describe contemporary real-world patterns of DCB and DES utilisation in patients undergoing PCI in Sri Lanka. Among 214 patients, DCB was used in 64 patients (29.9%) and DES in 150 patients (70.1%). Compared with previously published DCB and DES cohorts from Japan, Europe, the Middle East, and Asia, our study demonstrates several similarities but also notable differences in clinical presentation, prior PCI status, lesion characteristics, and target vessel distribution, reflecting variations in patient selection, healthcare systems, operator preference, and local PCI practice.

The clinical presentation of patients treated with DCB in our cohort was predominantly ACS, with STEMI and NSTEMI accounting for 67.2% of DCB-treated patients. Compared with previous DCB studies, this represents a greater use of DCB in acute presentations. In the PICCOLETO II trial [21], the majority of DCB-treated patients had stable angina (54.2%), with STEMI and NSTEMI accounting for only 10.3% and 21.1%, respectively. Similarly, the UK cohort (*n* = 148) [22] reported stable angina in 24.4% of DCB-treated patients, while DCB use was largely confined to carefully selected lesions. DCB is increasingly being considered in selected patients with ACS [23], reflecting growing clinical experience and emerging evidence supporting its use in appropriately prepared de novo lesions beyond its established indications of small-vessel disease and ISR [24].

Nevertheless, within our own cohort, DES remained the predominant revascularisation strategy for patients presenting with STEMI. Among DES-treated patients, 46.0% presented with STEMI compared with 29.7% of DCB-treated patients, this may reflect operator preference and lesion characteristics associated with acute presentations; however, these factors were not systematically assessed. This likely reflects the continued need for immediate mechanical stabilization in STEMI, particularly in the presence of significant thrombus burden, vessel recoil, flow-limiting dissections, or complex culprit lesions [8]. Thus, our findings suggest an evolution in DCB utilisation, with increasing application in selected ACS patients while DES was more frequently used than DCB among patients presenting with STEMI in this cohort.

### Clinical and anatomical factors influencing DCB selection

A notable finding in our study was the higher prevalence of previous PCI among DCB-treated patients compared with DES-treated patients (31.3% vs. 5.3%). This finding is not unexpected, as patients with a history of PCI are more likely to undergo repeat coronary intervention, and DCB is an established treatment option for coronary ISR, offering effective antiproliferative therapy without implantation of an additional metallic layer [8]. Although ISR is not specifically discussed in this comparison, the greater proportion of patients with previous PCI in the DCB group is consistent with contemporary DCB practice.

All 12 patients presenting with in-stent restenosis (ISR) in our cohort had previously undergone DES implantation and were treated with DCB angioplasty. This finding is particularly relevant in light of the DAEDALUS study, which reported a higher three-year risk of target lesion revascularisation with DCB than with repeat DES implantation in patients with DES-ISR (20.3% vs. 13.4%; HR 1.58, 95% CI 1.16–2.13) [25]. In contrast, no target lesion revascularisation was documented among the 12 DCB-treated ISR patients in our cohort during the six-month follow-up. Although this represents a favourable short-term observation, the small number of patients and limited follow-up preclude direct comparison with the three-year DAEDALUS findings or conclusions regarding the comparative effectiveness of DCB and repeat DES implantation. Nevertheless, our findings demonstrate the use of DCB to treat DES-ISR in routine clinical practice at our centre.

Lesion morphology also differed between treatment groups. Coronary calcification was documented in 6.7% of DES-treated patients. Calcification status was classified as “Not specified” in all 64 patients in the DCB group (100%), preventing a reliable assessment of its prevalence in this group or a valid comparison with the DES group. Contemporary lesion-selection practices may favour DES use for more complex or heavily calcified lesions when predictable stent expansion and sustained radial support are considered necessary [26]. However, calcification is not incompatible with DCB treatment; a Japanese comparative cohort [27] reported coronary calcification in 6.8% of DCB-treated lesions compared with 30.9% of DES-treated lesions, while the UK cohort (*n* = 148) [22] reported calcification in 78% of DCB-treated and 88% of DES-treated lesions. Successful DCB treatment requires careful lesion assessment and adequate lesion preparation to achieve an optimal acute angiographic result and facilitate effective drug delivery [28].

Procedural dissection was uncommon in our cohort, occurring in only one DCB-treated patient (1.6%) and none of the DES-treated patients. This differs from some European DCB cohorts, where angiographic dissections following balloon angioplasty were reported more frequently, including 28% in a UK DCB cohort (*n* = 170) [29] and 39% in the UK DCB cohort (*n* = 148) [22]. The lower frequency observed in our study may reflect careful lesion selection, meticulous lesion preparation, and operator preference for DES in lesions where vessel stability was considered uncertain. In the patient with type B dissection, lesion preparation involved multiple predilations using high-pressure balloon inflations, which may have contributed to the development of the dissection. Although classified as a procedural complication in our study, a type B dissection does not necessarily indicate procedural failure. Limited type A–B coronary dissections following DCB angioplasty may be considered acceptable when coronary flow is preserved and the final angiographic result is satisfactory, without necessarily requiring additional stenting [30, 31]. As DCB therapy relies on achieving optimal luminal expansion without permanent scaffolding, the assessment and management of angiographic dissections remain important considerations during DCB-based PCI [30, 31].

### DCB utilisation in small vessel disease, bifurcation lesions, and diffuse coronary disease

Beyond its established role in the treatment of ISR, contemporary evidence has expanded interest in DCB-based PCI for selected de novo coronary lesions, particularly small vessel disease, bifurcation lesions, and diffuse coronary disease. These anatomical subsets represent clinical scenarios where avoidance of permanent metallic implantation may provide potential advantages, including reduced metal burden, preservation of future revascularization options, and avoidance of complex stent implantation strategies. However, the effectiveness of DCB therapy remains highly dependent on appropriate lesion selection, adequate lesion preparation, and achievement of satisfactory angiographic results without significant recoil or flow-limiting dissection. The International DCB Consensus Group emphasized that DCB should be considered a lesion-specific treatment strategy rather than a universal replacement for DES, with careful assessment of anatomical suitability being essential [8].

Small vessel coronary disease represents the anatomical setting with the strongest randomised evidence supporting DCB use. In the BASKET-SMALL 2 trial [32], patients with de novo small vessel coronary lesions were randomised to DCB angioplasty or second-generation DES implantation. DCB demonstrated non-inferiority compared with DES for major adverse cardiac events, supporting DCB as an alternative strategy in appropriately selected small vessel lesions where stent implantation may be associated with increased restenosis risk due to limited vessel calibre. These findings have contributed to the expansion of DCB utilisation beyond ISR and provide a rationale for its application in selected lesions where vessel preservation is desirable. Although detailed vessel diameter classification was not available in our cohort, the use of DCB in contemporary Sri Lankan PCI practice reflects this broader shift toward individualised device selection based on anatomical and clinical considerations.

Bifurcation lesions represent another emerging area of interest for DCB-based strategies. Conventional DES treatment of bifurcation lesions may require complex stent techniques and may increase metallic burden, with potential risks including side branch compromise, restenosis, and difficulty with future interventions. The rationale for DCB use in bifurcation disease is based on the ability to deliver antiproliferative therapy without placing a permanent scaffold across the main vessel or side branch [8]. Recent clinical data have further explored this approach. The REC-CAGEFREE I bifurcation subgroup analysis [33] comparing DCB and DES strategies in non-complex bifurcation lesions demonstrated comparable device-oriented outcomes, although these findings remain exploratory and require confirmation in larger dedicated randomised studies. Similarly, a contemporary observational comparison of DCB-only versus DES strategies for coronary bifurcation lesions [34] reported favourable outcomes with DCB in selected patients, although treatment allocation was influenced by lesion complexity and operator judgement, limiting definitive conclusions regarding comparative efficacy. In our study, bifurcation-specific characteristics were not systematically recorded; however, the increasing adoption of DCB reflects the broader movement toward vessel-preserving PCI strategies in anatomically suitable lesions.

Diffuse coronary disease represents another important anatomical scenario where DCB therapy may offer theoretical advantages. Long and diffuse lesions frequently require extensive DES implantation, including long stented segments or overlapping stents, which may increase permanent metallic burden, delay vascular healing, and complicate future coronary interventions. A DCB-only strategy provides antiproliferative drug delivery while avoiding permanent scaffolding and may therefore be attractive in selected diffuse lesions, particularly when adequate lesion preparation allows satisfactory angiographic results without significant recoil or flow-limiting dissection [35]. The International DCB Consensus Group has highlighted long lesions and diffuse disease as potential areas where a “leave nothing behind” approach may be advantageous, although comparative randomised evidence remains less established than for small vessel disease and ISR [8]. Real-world registries, such as the DEB-DRAGON registry, have demonstrated the feasibility of DCB treatment in selected de novo and complex coronary lesions, supporting its potential application beyond traditional indications [36].

In our cohort, diffuse coronary disease was identified in 18.8% of lesions treated with DCB and 11.3% of lesions treated with DES (*p* = 0.025). Diffuse coronary disease was more frequently recorded among DCB-treated patients than DES-treated patients in our cohort. However, this finding should be interpreted cautiously, as diffuse disease was assessed by visual angiographic classification, and detailed measures such as lesion length, reference vessel diameter, plaque burden, and intravascular imaging findings were not systematically available. Therefore, the observed association does not establish that diffuse disease itself influenced the choice of DCB over DES. The clinical and anatomical factors underlying this treatment selection could not be determined from the available data. This difference may reflect, in part, the clinical appeal of a stent-free strategy in diffuse disease, where avoidance of extensive or overlapping stent implantation may be desirable.

Although our study did not systematically quantify lesion length, vessel diameter, plaque morphology, or diffuse disease severity using intravascular imaging or quantitative coronary angiography, the observed pattern of DCB utilisation provides important insight into real-world PCI decision-making. The preferential use of DCB in selected patients with previous PCI and diffuse coronary involvement, together with continued DES preference in acute presentations and anatomically complex lesions requiring mechanical support, suggests that contemporary PCI practice is moving toward a more individualised approach rather than a single device strategy. Future prospective studies incorporating lesion length, bifurcation classification, quantitative angiographic assessment, and intravascular imaging will be important to define which anatomical subsets derive the greatest benefit from DCB-based PCI.

### Target vessel distribution and lesion selection

The distribution of target vessels in our cohort was comparable with previous studies, with LAD involvement being common among both treatment groups internationally. Studies from Qatar, China, Japan, and the PICCOLETO trial demonstrated similar predominance of LAD lesions, suggesting that vessel location alone may not be a major determinant of DCB versus DES selection. Instead, lesion complexity, vessel diameter, previous stent history, and procedural considerations are likely more influential factors guiding device choice.

### Short-term clinical outcomes and comparison with contemporary evidence

Early clinical outcomes in our cohort were infrequent in both treatment groups. By 6 months, all-cause mortality was observed in 3 DCB patients (4.7%) and 3 DES patients (2.0%), with heart failure accounting for the majority of cardiac deaths in both groups. Owing to the retrospective study design, relatively small sample size, low event rates, and lack of adjustment for baseline differences, these findings should not be interpreted as evidence of comparative safety or efficacy between DCB and DES. Nevertheless, few outcome events were observed during the available follow-up and is consistent with previous studies demonstrating acceptable short- to mid-term outcomes with contemporary DCB therapy in appropriately selected patients. Large randomised trials such as BASKET-SMALL 2 [32], PICCOLETO II [21], and RESTORE SVD [37] have reported clinical outcomes comparable to contemporary DES in selected lesion subsets, particularly small vessel disease, although these results cannot be directly extrapolated to our broader real-world cohort because treatment selection was based on operator discretion and lesion characteristics.

### Implications for DCB practice in the Sri Lankan context

The differences observed between our cohort and international studies highlight the importance of considering local practice patterns when interpreting DCB utilisation. While DCB therapy has historically been restricted to specific indications, increasing clinical experience and improvements in device technology have expanded its application. Our findings suggest that DCB is being incorporated into routine PCI practice in Sri Lanka, including selected ACS presentations and patients with previous PCI, while DES continues to remain the dominant strategy, particularly in acute presentations and lesions requiring mechanical support. Nevertheless, DCB utilisation in Sri Lanka may be influenced by individual operator preference, clinical judgement, lesion characteristics, resource constraints, and device availability. To date, the study by Herath et al. appears to be the only published study specifically reporting clinical outcomes following DCB angioplasty in Sri Lanka, providing an important local perspective on its use in clinical practice [10]. Therefore, DCB utilisation at our centre may reflect local operator practice rather than national utilisation patterns.

### Strengths and limitations

To our knowledge, this is among the first comparative real-world studies from Sri Lanka evaluating DCB vs. DES in routine clinical practice, thereby contributing valuable regional evidence from a setting where comparative data remain limited. It provides contemporary real-world data reflecting routine clinical practice and includes a broad spectrum of patients treated with either DCB or DES. Unlike many previous studies that have focused on selected lesion subsets, our cohort encompasses both de novo lesions and ISR, allowing evaluation of device selection across diverse clinical scenarios. In addition to procedural and clinical outcomes, the study incorporates prospectively collected patient-reported outcomes, providing a patient-reported symptom and functional status.

This study has several limitations. In this retrospective single-centre observational design, DCB or DES selection was individualised according to patient characteristics, lesion morphology, clinical presentation, and operator judgement. However, the absence of randomised treatment allocation and differences in clinical presentation between the groups, particularly ACS versus non-ACS presentations, introduces potential confounding by indication and limits causal comparisons between treatment groups. The relatively small sample size, short follow-up period, and low number of adverse clinical events limited meaningful adjusted outcome analyses; therefore, clinical outcome comparisons should be interpreted cautiously. Additionally, the single-centre design may limit generalisability to other populations. The interpretation of certain lesion characteristics was limited by how these characteristics were documented in the available procedural records, particularly lesion diffuseness and calcification. This may have affected the assessment and comparison of lesion characteristics between the DCB and DES groups. ​

Patient-reported outcomes were assessed using modified SAQ domains rather than the validated SAQ-7 instrument and should therefore be interpreted with caution when compared with studies using validated tools.

## Conclusion

This single-centre observational study provides real-world evidence on DCB and DES utilisation in PCI practice in Sri Lanka. DCBs were used particularly in patients with previous coronary revascularisation, small-vessel and branch-vessel disease, and elective PCI. Previous revascularisation was independently associated with higher odds of DCB use, while acute PCI was associated with lower odds than elective PCI. Few clinical events occurred during follow-up, with no statistically significant differences in short-term outcomes or patient-reported measures. These findings suggest that DCB therapy is an integrated component of PCI practice in this Sri Lankan centre, extending beyond ISR to selected de novo and acute coronary presentations. Overall, DCB and DES appear to serve complementary roles in individualised, lesion- and patient-specific treatment. Given the non-randomised design, small sample size, low event rate, and short follow-up, these findings should be considered descriptive and hypothesis-generating.

## Data Availability

The anonymised dataset used and analysed during the current study is available from the corresponding author upon reasonable request.

## Abbreviations

CAD: Coronary artery disease
PCI: Percutaneous coronary intervention
DES: Drug-eluting stent
DAPT: Dual antiplatelet therapy
DCB: Drug-coated balloon
ISR: In-stent restenosis
PRO: Patient reported outcomes
CABG: Coronary artery bypass graft
CKD: Chronic kidney disease
ACS: Acute coronary syndrome
EF: Ejection fraction
STEMI: ST-elevation myocardial infarction
NSTEMI: Non-ST-elevation myocardial infarction
HF: Heart failure
AKI: Acute kidney injury
TLR: Target lesion revascularisation
TVR: Target vessel revascularisation
SAQ: Seattle angina questionnaire
SD: Standard deviation
IQR: Interquartile range
UA: Unstable angina
LAD: Left anterior descending
RCA: Right coronary artery
OR: Odds ratio
CI: Confidence interval
